# Impact of Donor Age on the Osteogenic Supportive Capacity of Mesenchymal Stromal Cell-Derived Extracellular Matrix

**DOI:** 10.3389/fcell.2021.747521

**Published:** 2021-10-05

**Authors:** Marta S. Carvalho, Laura Alves, Isabel Bogalho, Joaquim M. S. Cabral, Cláudia L. da Silva

**Affiliations:** ^1^Department of Bioengineering and IBB-Institute for Bioengineering and Biosciences, Instituto Superior Técnico, Universidade de Lisboa, Lisbon, Portugal; ^2^Associate Laboratory i4HB-Institute for Health and Bioeconomy, Instituto Superior Técnico, Universidade de Lisboa, Lisbon, Portugal

**Keywords:** aging, cell-derived extracellular matrix, decellularization, mesenchymal stromal cells (MSC), osteogenic differentiation

## Abstract

Mesenchymal stromal cells (MSC) have been proposed as an emerging cell-based therapeutic option for regenerative medicine applications as these cells can promote tissue and organ repair. In particular, MSC have been applied for the treatment of bone fractures. However, the healing capacity of these fractures is often compromised by patient’s age. Therefore, considering the use of autologous MSC, we evaluated the impact of donor age on the osteogenic potential of bone marrow (BM)-derived MSC. MSC from older patients (60 and 80 years old) demonstrated impaired proliferative and osteogenic capacities compared to MSC isolated from younger patients (30 and 45 years old), suggesting that aging potentially changes the quantity and quality of MSC. Moreover, in this study, we investigated the capacity of the microenvironment [i.e., extracellular matrix (ECM)] to rescue the impaired proliferative and osteogenic potential of aged MSC. In this context, we aimed to understand if BM MSC features could be modulated by exposure to an ECM derived from cells obtained from young or old donors. When aged MSC were cultured on decellularized ECM derived from young MSC, their *in vitro* proliferative and osteogenic capacities were enhanced, which did not happen when cultured on old ECM. Our results suggest that the microenvironment, specifically the ECM, plays a crucial role in the quality (assessed in terms of osteogenic differentiation capacity) and quantity of MSC. Specifically, the aging of ECM is determinant of osteogenic differentiation of MSC. In fact, old MSC maintained on a young ECM produced higher amounts of extracellularly deposited calcium (9.10 ± 0.22 vs. 4.69 ± 1.41 μg.μl^–1^.10^–7^ cells for young ECM and old ECM, respectively) and up-regulated the expression of osteogenic gene markers such as *Runx2* and *OPN.* Cell rejuvenation by exposure to a functional ECM might be a valuable clinical strategy to overcome the age-related decline in the osteogenic potential of MSC by recapitulating a younger microenvironment, attenuating the effects of aging on the stem cell niche. Overall, this study provides new insights on the osteogenic potential of MSC during aging and opens new possibilities for developing clinical strategies for elderly patients with limited bone formation capacity who currently lack effective treatments.

## Introduction

Mesenchymal stromal cells (MSC) have been described as a cell population of multipotent stem/progenitor cells with the ability to proliferate and differentiate into multiple lineages, including adipogenic, chondrogenic, and osteogenic (Pittenger et al., 1999, 2019). In addition, MSC are known for their immunomodulatory properties, which make these cells a promising tool for regenerative medicine applications (Pittenger et al., 2019). In particular, several studies have supported the potential application of *ex vivo* expanded MSC, mostly of autologous origin (Rapp et al., 2018), to improve the current clinical practices for repairing large bone defects (Schmitt et al., 2012; Perez et al., 2018). However, the healing capacity of these fractures is often compromised by patients’ age and comorbidities, including diabetes and osteoporosis (Vester et al., 2014; Huang et al., 2016). In this context, it is important to understand the impact of donor age on the therapeutic potential of MSC, when considering the use of autologous MSC to treat non-healing fractures. In fact, it was demonstrated that aging decreased the proliferative potential of bone marrow (BM)-derived MSC and accelerated cell apoptosis and senescence (Stenderup et al., 2003). Overall, the quantity and quality of BM MSC decrease with age, compromising the feasibility and success of exogenous MSC therapies (i.e., administration of *ex vivo* expanded cells) in elderly patients (Block et al., 2017). Importantly, the impact on donor age is not restricted to cells themselves but also to their microenvironment that is known to affect cell function (Asumda, 2013).

Decellularized cell-derived extracellular matrix (ECM) has been used as a strategy to create a native cellular microenvironment on scaffolds by combining biological and physical cues (Hoshiba et al., 2010; Carvalho et al., 2019b). Cell-derived ECM consists of a complex network of fibrillary proteins, matrix macromolecules, and growth factors that mimic the composition and organization of native microenvironment (Carvalho et al., 2019b). ECM proteins are secreted by cultured cells and are retained after decellularization (Hoshiba et al., 2006; Carvalho et al., 2019b). This decellularized ECM can control cell fate through topography, substrate stiffness, and biochemical signaling (Nicolas et al., 2020). Studies have reported promising results using decellularized cell-derived ECM to enhance the bioactivity and osteogenic potential of scaffolds for bone tissue engineering applications (Liao et al., 2010; Lin et al., 2012; Carvalho et al., 2019c; Silva et al., 2020). Previous results from our group have shown that ECM derived from cultured BM MSC can mimic the stem/stromal cell niche facilitating proliferation and improving osteogenic differentiation of MSC (Carvalho et al., 2019b, c; Silva et al., 2020). Although much progress has been made in understanding the clinical potential of cell-derived ECM, the impact of donor age on the osteogenic supportive capacity of MSC-derived ECM is still not completely understood. Seminal work by Conboy et al. (2005) suggested that the microenvironment of a young animal can promote successful tissue regeneration, whereas that of an older animal fails to promote regeneration. Furthermore, these authors demonstrated that modulation of systemic factors can reverse the decline of tissue regenerative potential, suggesting that age-related changes in cell niche can affect cells regenerative potential (Conboy et al., 2005). Nevertheless, the possible mechanisms underlying the rejuvenation potential of ECM need to be elucidated. Therefore, this led us to investigate whether a young ECM could increase the number and promote the osteogenic capacity of aged MSC. Cell rejuvenation by exposure to a functional ECM might be a critical step for multiple potential therapeutic applications.

In this context, we hypothesized that decellularized ECM derived from young MSC can enhance different cellular functions, such as proliferation and osteogenic differentiation potential. To this end, in this study, we evaluated morphology, growth kinetics, and osteogenic differentiation activity of BM MSC obtained from donors of different age groups (30 and 45 years old and 60 and 80 years old). Then, we evaluated the impact of aging in the capacity of MSC-derived ECM to restore the *in vitro* proliferative and osteogenic activity of aged MSC. These results might be especially important when developing autologous cell-based therapies for elderly patients, namely, for those with limited bone formation capacity and with the stem/stromal cell niche impaired by aging.

## Materials and Methods

### Cell Culture

The human MSC used were part of the cell bank available at the Stem Cell Engineering Research Group, Institute for Bioengineering and Biosciences (iBB) at Instituto Superior Técnico (IST). MSC were previously isolated according to protocols previously established at iBB-IST (Carvalho et al., 2018, 2019a). Samples were obtained from Instituto Português de Oncologia Francisco Gentil, Lisboa, and Centro Clínico da GNR, Lisboa, under collaboration agreements with iBB-IST. All human samples were obtained from healthy donors after written informed consent according to Directive 2004/23/EC of the European Parliament and of the Council of March 31, 2004, on setting standards of quality and safety for the donation, procurement, testing, processing, preservation, storage, and distribution of human tissues and cells (Portuguese Law 22/2007, June 29), with the approval of the Ethics Committee of the respective clinical institution. Isolated cells were kept frozen in liquid/vapor nitrogen tanks until further use. MSC were obtained from BM and femur of young (30 and 45 years old; referred to as “*young*” MSC) and old donors (60 and 80 years old; referred to as “*old*” MSC). MSC were thawed and plated on T-75 flasks using low-glucose Dulbecco’s modified Eagle’s medium (DMEM; Gibco, Grand Island, NY, United States) supplemented with 10% fetal bovine serum (FBS MSC qualified: Gibco) and 1% antibiotic-antimycotic (Gibco) and kept at 37°C, 5% CO_2_ in a humidified atmosphere. Medium renewal was performed every 3 days. Cells between passages 2 and 4 were used in this study. Two independent donors from each age group (young vs. old) were used in this study. Three independent experiments were performed per donor with three technical replicates.

### Immunophenotypic Analysis

Upon cell isolation based on plastic adherence, MSC from different age donors were analyzed by flow cytometry using a panel of mouse anti-human monoclonal antibodies (Biolegend, San Diego, CA, United States) for the expression of CD73, CD90, CD105, CD34, and CD45. Briefly, cells and controls were incubated with each antibody for 20 min in the dark at room temperature, washed with phosphate-buffered saline solution (PBS; Gibco), and fixed using a solution of 2% paraformaldehyde (PFA; Sigma-Aldrich, St. Louis, MO, United States). A minimum of 10,000 events were acquired for each sample. Flow cytometric analysis was performed using FACScalibur flow cytometer (Becton Dickinson, Franklin Lakes, NJ, United States) and CellQuest^TM^ software (Becton Dickinson) was used for acquisition. For data analysis, Flowing Software (University of Turku, Finland) was used.

### Multilineage Differentiation Assays

To investigate the multipotency of MSC from different age donors, *in vitro* multilineage differentiation studies (adipogenic, chondrogenic, and osteogenic lineages) were performed.

#### Adipogenic Differentiation Potential

For adipogenic differentiation, MSC were cultured at 3,000 cells/cm^2^ on 24-well plates with DMEM + 10% FBS. At 80% confluence, adipogenesis was induced using the StemPro^®^ Adipogenesis Differentiation Kit (Gibco). Medium was changed every 3–4 days for 21 days. The presence of adipocytes was verified by staining for triglycerides with Oil Red O (Sigma-Aldrich), an indicator of intracellular lipids accumulation. Briefly, cells were washed with PBS and fixed with 4% PFA for 20 min. Then, cells were washed with distilled water and incubated with Oil Red O solution (0.3% in isopropanol) at room temperature for 1 h.

#### Chondrogenic Differentiation Potential

For chondrogenic differentiation, MSC were cultured as cell aggregates. Cells were plated as small droplets (10 μl) with a cell density of 1 × 10^7^ cells/ml on ultra-low attachment culture plates. After 30 min, StemPro^®^ Chondrogenic Differentiation Kit (Gibco) was added to the culture. Medium was changed every 3–4 days for 21 days. These cultures were stained with Alcian Blue (Sigma-Aldrich) for assessing synthesis of proteoglycans by chondrocytes. Cells were washed with PBS and fixed with 4% PFA for 20 min. Then, cells were washed with distilled water and incubated with 1% Alcian Blue solution at room temperature for 1 h.

#### Osteogenic Differentiation Potential

For osteogenic differentiation, MSC were cultured at 3,000 cells/cm^2^ on 24-well plates with DMEM + 10% FBS. At 80% confluence, cells were incubated with osteogenic differentiation medium composed by DMEM supplemented with 10% FBS, 10 mM β-glycerophosphate (Sigma-Aldrich), 10 nM dexamethasone (Sigma-Aldrich), 50 μg/ml ascorbic acid (Sigma-Aldrich), and 1% antibiotic–antimycotic. Medium was changed every 3–4 days for 21 days. Cultures were stained with alkaline phosphatase (ALP) and von Kossa stainings to identify ALP activity and mineralization, respectively, indicating the presence of active osteoblasts. Cells were washed with PBS and fixed with 4% PFA for 20 min. Then, cells were rinsed with milliQ water and incubated with a Fast Violet solution (Sigma-Aldrich) and Naphthol AS-MX phosphate alkaline solution (Sigma-Aldrich) in a final concentration of 4% for 45 min at room temperature in the dark. Cells were washed and then incubated with a silver nitrate solution (Sigma-Aldrich) for 30 min at room temperature in the dark (von Kossa staining). Alizarin Red staining was also performed to visualize calcium deposits. Cells were stained with a 2% Alizarin Red solution (Sigma-Aldrich) by incubation for 1 h at room temperature.

### Proliferation Assays

Mesenchymal stromal cells from young and old donors were plated onto 12-well plates at 3,000 cells/cm^2^ using DMEM + 10% FBS as growth medium. Cells were kept at 37°C and 5% CO_2_ in a humidified atmosphere, and culture medium was changed every 3–4 days. At days 2, 4, 7, and 10, cells were harvested using a solution of 0.05% trypsin (Gibco) and counted using the Trypan Blue exclusion method (Gibco) to determine cell growth curves.

### Cell Morphology

Mesenchymal stromal cells from young and old donors were seeded on 24-well plates, and cell morphology was assessed after 4, 7, and 10 days of culture. Cells were washed twice with PBS, fixed with 4% PFA for 20 min, and then permeabilized with a 0.1% Triton X-100 solution (Sigma-Aldrich) for 10 min. After permeabilization, cells were incubated with phalloidin-TRITC (Sigma-Aldrich; 2 μg/ml) for 45 min in the dark. Then, cells were washed twice with PBS and counterstained with 4′,6-diamidino-2-phenylindole (DAPI; Sigma-Aldrich; 1.5 μg/ml) for 5 min and washed with PBS. Cells were imaged by fluorescence microscope (Leica DMI3000B, Wetzlar, Germany).

### Decellularized Cell-Derived Extracellular Matrix Preparation and Characterization

To prepare decellularized ECM derived from MSC with different ages, young MSC and old MSC were cultured at 3,000 cells/cm^2^. MSC culture was maintained with DMEM + 10% FBS and medium was renewed every 3–4 days. After reaching confluency, between days 7 and 10, medium was discarded and cells were washed with PBS. Decellularization was performed according to previously established protocols (Carvalho et al., 2019b, c; Silva et al., 2020). Briefly, cells were incubated with a solution of 0.5% Triton X-100 containing 20 mM NH_4_OH (Sigma-Aldrich) in PBS for 5 min. After microscopic confirmation of complete cell lysis and presence of ECM on the surface of the wells, MSC-derived ECM from young MSC (young ECM) and old MSC (old ECM) were gently washed using PBS and air dried under the laminar flow hood.

To evaluate the protein components and distribution patterns of the different ECM, immunofluorescent staining was performed. Collagen I, fibronectin, and laminin were stained. After decellularization treatment, MSC-derived ECM was washed with PBS and fixed with 4% PFA for 20 min at room temperature. Then, cell-derived ECM was washed three times with 1% bovine serum albumin (BSA) solution for 5 min. Samples were blocked with a solution of 0.3% Triton X-100, 1% BSA, and 10% donkey serum (Sigma-Aldrich) in PBS at room temperature for 45 min. Primary antibodies including rabbit anti-human collagen I, rabbit anti-human laminin, and mouse anti-human fibronectin (10 μg/ml in 0.3% Triton X-100, 1% BSA and 10% donkey serum; ThermoFisher Scientific, Waltham, MA, United States) were added into the samples followed by incubation overnight at 4°C. After washing with 1% BSA solution, goat anti-rabbit IgG Alexa Fluor 546 and goat anti-mouse IgG Alexa Fluor 488 secondary antibodies (dilution 1:200 in 1% BSA solution; ThermoFisher Scientific) were added into the samples and incubated in the dark for 1 h at room temperature. Finally, the cell nuclei were counterstained with DAPI (1.5 μg/ml) for 5 min and then washed with PBS. The staining was imaged by fluorescence microscopy (Leica DMI3000B) and recorded by an attached digital camera.

### Mesenchymal Stromal Cells Culture on Mesenchymal Stromal Cells-Derived Extracellular Matrix From Young and Old Donors

Young MSC and old MSC were cultured at 3,000 cells/cm^2^ on 12 well-plates or 24 well-plates coated with decellularized ECM derived from young MSC (young ECM) or old MSC (old ECM) according to section “Decellularized Cell-Derived Extracellular Matrix Preparation and Characterization”. Proliferation and morphology assays were performed according to sections “Proliferation Assays” and “Cell Morphology.”

### Assessment of Mesenchymal Stromal Cells Osteogenic Differentiation

#### Alkaline Phosphatase Activity Assay

Alkaline phosphatase activity was quantified using a colorimetric ALP kit (BioAssays Systems, Hayward, CA, United States) according to the manufacturer’s protocol. Samples were washed with PBS and incubated in the lysis buffer (0.1% Triton X-100 solution) by shaking for 30 min at room temperature. The lysate was added to p-nitrophenyl phosphate solution (10 mM) provided with the ALP kit. The absorbance was measured at 405 nm and normalized to the total number of cells in each sample after 21 days under osteogenic differentiation conditions.

#### Calcium Assay

To determine the calcium content, samples were washed twice with PBS and extracted in 0.5 M HCl solution (Sigma-Aldrich). Calcium was removed from the cellular component by shaking overnight at 4°C. Supernatants were used for calcium quantification according to the manufacturer’s instructions contained in the calcium colorimetric assay kit (Sigma-Aldrich). Absorbance at 575 nm was measured for each condition and normalized to the total number of cells after 21 days under osteogenic differentiation conditions.

#### Immunocytochemistry Analysis

Mesenchymal stromal cells were plated on 24-well plates and osteogenic differentiation was induced. After 21 days of culture, cells were washed with PBS and fixed with 4% PFA for 20 min at room temperature. Cells were then washed three times with 1% BSA for 5 min, permeabilized, and blocked with a solution of 0.3% Triton X-100, 1% BSA, and 10% donkey serum in PBS at room temperature for 45 min. Primary antibodies including rabbit anti-human osteopontin (OPN; Abcam, Cambridge, United Kingdom) and rabbit anti-human osteocalcin (OC; Thermo Fisher Scientific; 10 μg/ml in 0.3% Triton X-100, 1% BSA, 10% donkey serum solution) were added followed by overnight incubation at 4°C. After washing with 1% BSA in PBS, goat anti-rabbit IgG Alexa Fluor 546 (1:200 in 1% BSA solution; ThermoFisher Scientific) was used as secondary antibody and incubated in the dark for 1 h at room temperature. Finally, the cell nuclei were counterstained with DAPI (1.5 μg/ml) for 5 min and then washed with PBS. The staining was imaged by fluorescence microscopy (Leica DMI3000B).

#### RNA Extraction and Quantitative Real-Time PCR Analysis

Total RNA was extracted using the RNeasy Mini Kit (QIAGEN, Hilden, Germany). cDNA was synthesized from 10 ng of total RNA using iScript^TM^ Reverse Transcription Supermix (Bio-Rad, Hercules, CA, United States). Reaction mixtures were incubated in a thermal cycler (Veriti 96-well thermal cycler; Applied Biosystems, Foster City, CA, United States) for 5 min at 25°C, 30 min at 42°C, and 5 min at 85°C and then were maintained at 4°C. The sequences of the specific primer sets used are given in Table 1. The quantitative reverse transcription-polymerase chain reaction was performed using NZYSpeedy qPCR Green Master Mix (2x), Rox Plus (NZYTech, Portugal), and StepOnePlus real-time PCR system (Applied Biosystems). All reactions were carried out at 95°C for 10 min, followed by 40 cycles at 95°C for 15 s and 60°C for 1 min; all reactions were performed in triplicate. Glyceraldehyde 3-phosphate dehydrogenase was used as internal control to normalize differences in total RNA levels in each sample. A threshold cycle (Ct) was observed in the exponential phase of amplification, and quantification of relative expression levels was performed with the use of standard curves for target genes and endogenous control. Geometric means were used to calculate the ΔΔCt values and expressed as 2^–ΔΔCt^. The mean values from triplicate analysis were compared. The values obtained for MSC at day 0 (no differentiation) were set as 1 and were used to calculate the fold difference in the target gene.

**TABLE 1:** Sequences of primers used for qRT-PCR analysis.

Gene	Primer sequence
GAPDH	For: 5′ AAC AGC GAC ACC CAC TCC TC Rev: 5′ CAT ACC AGG AAA TGA GCT TGA CAA
Col I	For: 5′ CAT CTC CCC TTC GTT TTT GA Rev: 5′ CCA AAT CCG ATG TTT CTG CT
Runx2	For: 5′ AGA TGA TGA CAC TGC CAC CTC TG Rev: 5′ GGG ATG AAA TGC TTG GGA ACT
ALP	For: 5′ ACC ATT CCC ACG TCT TCA CAT TT Rev: 5′ AGA CAT TCT CTC GTT CAC CGC C
OPN	For: 5′ ATG AGA TTG GCA GTG ATT Rev: 5′ TTC AAT CAG AAA CCT GGA A
OC	For: 5′ TGT GAG CTC AAT CCG GCA TGT Rev: 5′ CCG ATA GGC CTC CTG AAG C

### Statistical Analysis

Three independent experiments were performed for each donor. Moreover, each experiment was conducted in triplicate with replicates. The statistical analysis of the data was performed using one-way and two-way ANOVA followed by Tukey’s multiple comparisons test. Student’s *t*-test was used to compare results of old MSC and young MSC. GraphPad Prism version 6 software was used in the analysis. Data were considered to be significant when *p*-values obtained were <0.05 (95% confidence intervals, ^∗^*p* < 0.05).

## Results

### Characterization of Mesenchymal Stromal Cells From Young and Old Donors

**FIGURE 1:**
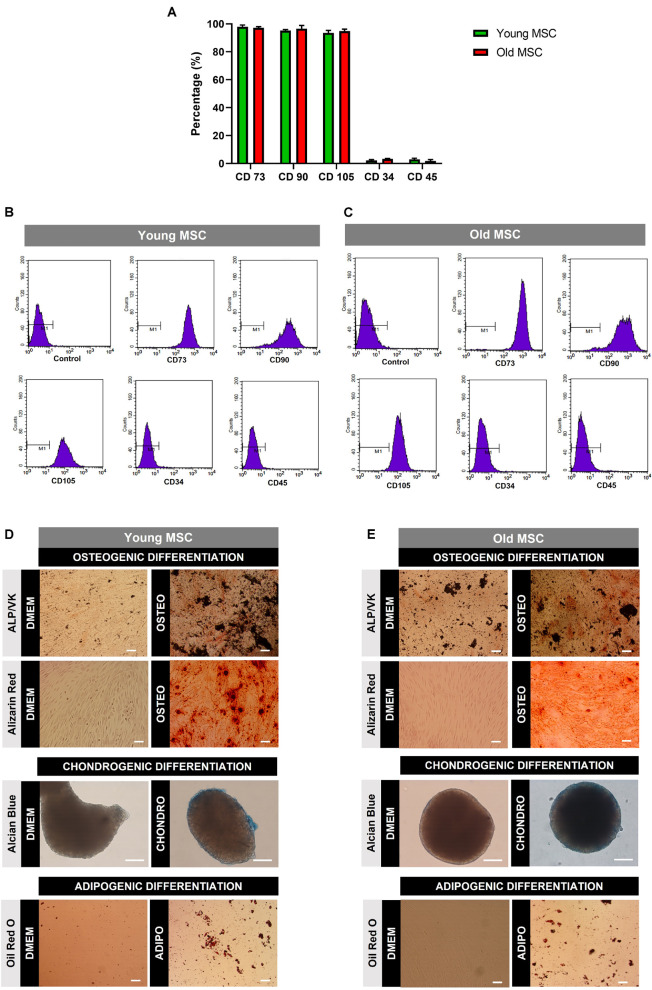
Characterization of mesenchymal stromal cells (MSC) obtained from young and old donors. **(A)** Surface marker expression by MSC isolated from young donors (30 and 45 years old) and old donors (60 and 80 years old) by flow cytometry. MSC were positive for CD73, CD90, and CD105 but negative for CD34 and CD45. **(B)** Flow cytometry plots of surface marker expression by young MSC. **(C)** Flow cytometry plots of surface marker expression by old MSC. **(D)** Multilineage differentiation analysis of MSC from young donors. **(E)** Multilineage differentiation analysis of MSC from old donors. Cells were cultured under osteogenic, chondrogenic, and adipogenic differentiation media for 21 days. DMEM: cells before differentiation (day 0), OSTEO, CHONDRO, and ADIPO: cells differentiated at day 21. Scale bars, 100 μm.

Human MSC from old (60 and 80 years old) and young (30 and 45 years old) donors were characterized by flow cytometry and multilineage differentiation assays (Figure 1 and Supplementary Figure 1). Results revealed that cells from old and young donors expressed the expected markers CD73, CD90, and CD105 but did not express the CD34 hematopoietic marker and the leukocyte common antigen CD45 (Figures 1A–C), according to standard identity criteria established for MSC in Dominici et al. (2006). Despite the age disparity, no significant changes in the expression of the cell markers were found (Figure 1A). Moreover, MSC isolated from older and younger donors were able to successfully differentiate into osteogenic, chondrogenic, and adipogenic lineages as observed in Figures 1D,E.

### Effect of Donor Age on Mesenchymal Stromal Cells Morphology and Growth Kinetics

**FIGURE 2:**
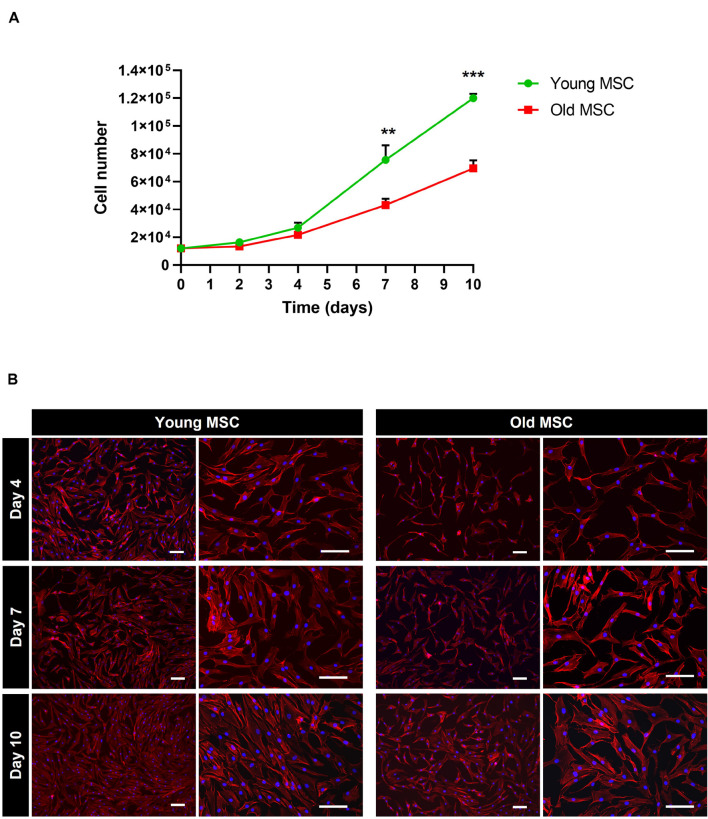
Impact of donor age on the proliferation and morphology of mesenchymal stromal cells (MSC). **(A)** Comparison of the *in vitro* proliferation of MSC from young (30 and 45 years old) and old (60 and 80 years old) donors. **(B)** Cell morphology of young MSC and old MSC assessed by DAPI-Phalloidin staining at day 4, 7, and 10. Values are expressed as mean ± SD (two donors/group, three independent experiments/donor); **p < 0.01; ***p < 0.001; Scale bars, 100 μm.

In what concerns cell proliferation, cells from younger donors presented a significantly higher rate in comparison to elderly donors. After 7 days of culture, a significant increase in cell numbers was observed for young MSC compared to old MSC (*p* < 0.01; Figure 2A). In fact, young MSC presented a fold expansion of 6.3, reaching a cell number of (0.75 ± 0.10) × 10^5^, whereas old MSC presented a fold expansion of 3.6, reaching a cell number of (0.43 ± 0.03) × 10^5^ (*p* < 0.01; Figure 2A). After 10 days in culture, young MSC presented a statistically significant increase in proliferation, reaching a cell number of (1.2 ± 0.03) × 10^5^ (fold expansion of 10), whereas old MSC achieved around half that number (0.69 ± 0.06) × 10^5^ cells (fold expansion of 5.8; *p* < 0.001). Despite this, cells from both ages exhibited similar spindle shaped morphology (Figure 2B). Taken together, these results suggest that the proliferative capacity of MSC *in vitro* decreases with the increase of donor age.

### Age-Related Changes in Mesenchymal Stromal Cells Cultured Under Osteogenic Conditions

**FIGURE 3:**
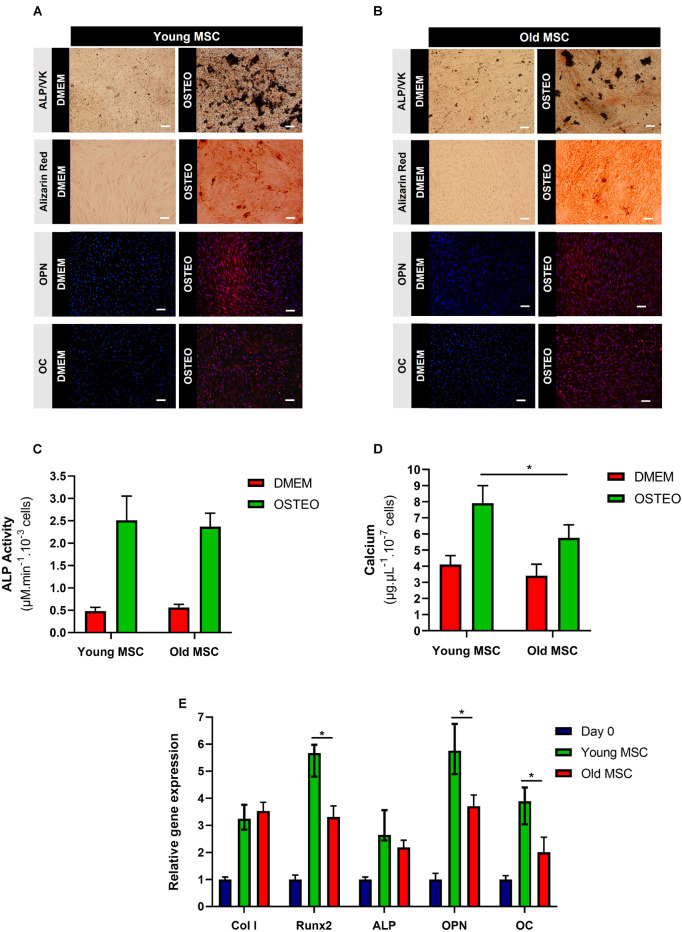
Impact of donor age on mesenchymal stromal cells (MSC) osteogenic differentiation *in vitro*. **(A)** Alkaline phosphatase (ALP), von Kossa (VK), Alizarin Red, osteopontin (OPN), and osteocalcin (OC) stainings of young MSC (30 and 45 years old) after 21 days of culture under osteogenic conditions. **(B)** ALP, VK, Alizarin Red, OPN, and OC stainings of old MSC (60 and 80 years old) after 21 days of culture under osteogenic conditions. DAPI was used to counterstain the cell nuclei in blue. DMEM: cells before differentiation (day 0), OSTEO: cells after 21 days of culture under osteogenic differentiation conditions. **(C)** ALP activity of young and old MSC after 21 days under osteogenic differentiation conditions. **(D)** Calcium deposition quantification of young and old MSC. **(E)** Osteogenic gene expression by young MSC and old MSC after 21 days under osteogenic differentiation conditions (*Col I*, *Runx2*, *ALP*, *OPN*, *OC*). Results were normalized to the endogenous control GAPDH and presented as fold change expression relative to undifferentiated MSC (day 0). Values are expressed as mean ± SD (two donors/group, three independent experiments/donor); *p < 0.05; Scale bars, 100 μm.

To evaluate the impact of donor age on the *in vitro* osteogenic potential of MSC, quantification of ALP activity and calcium deposition were evaluated, as well as osteogenic gene expression analysis, after 21 days of culture under osteogenic differentiation conditions. ALP staining (reddish areas; Figures 3A,B) and its respective quantification (Figure 3C) confirmed osteoprogenitor activity in MSC from both age donors, without statistically significant differences. Alizarin Red and von Kossa stainings confirmed the presence of calcium deposits in young and old MSC after culture under osteogenic induction conditions, demonstrating the successful differentiation of MSC from different ages into osteoblasts (Figures 3A,B). Immunocytochemistry staining of OPN and OC also confirmed that aged MSC successful differentiated into an osteogenic lineage (Figure 3B). However, MSC derived from younger donors presented the most abundant reaction of Alizarin Red and von Kossa, as well as the highest concentration of calcium (Figures 3A–D). In fact, a statistically significant enhancement in calcium accumulation (around 38%) was observed in MSC derived from young donors compared to older donors (*p* < 0.05; Figure 3D). These differences were corroborated by the assessment of gene expression levels of osteogenic markers, such as *Col I*, *Runx2*, *ALP*, *OPN*, and *OC*. Cells from both age donor groups (young and old MSC) up-regulated the expression of osteogenic gene markers compared to the control (undifferentiated cells at day 0). Statistically significant differences in the expression levels of *Runx2*, *OPN*, and *OC* were observed between young and old MSC (*p* < 0.05; Figure 3E). Although a slight increase in *ALP* gene expression was observed in young MSC compared to old MSC, such difference was not considered statistically significant in the conditions of our study. Overall, significant differences between young and elderly donors were observed concerning the levels of mineralization and osteogenic gene expression.

### Effects of Donor Age on the Proliferation Support Capacity of Mesenchymal Stromal Cells-Derived Extracellular Matrix

**FIGURE 4:**
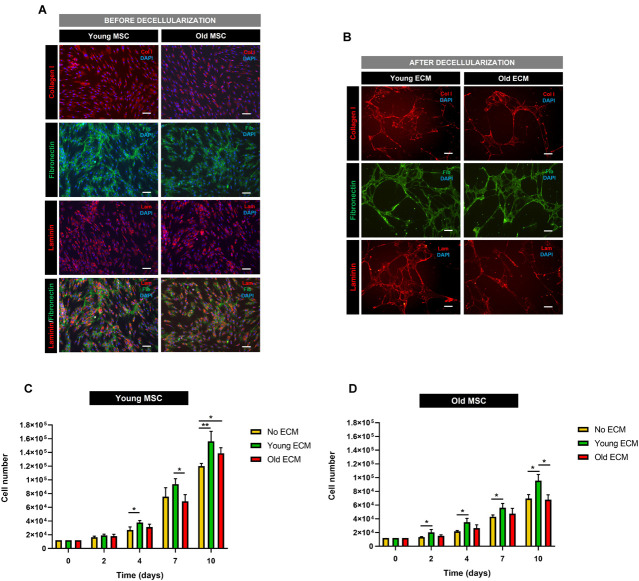
Impact of donor age on the biochemical characterization and proliferation support capacity of mesenchymal stromal cell-derived extracellular matrix. **(A)** Characterization of mesenchymal stromal cells (MSC) from young and old donors before decellularization. **(B)** Characterization of MSC extracellular matrix (ECM) from young and old donors after decellularization. Immunofluorescent staining images of collagen I, fibronectin, and laminin. **(C)** Cell proliferation assay of young MSC cultured on young ECM, old ECM, and without ECM. **(D)** Cell proliferation assay of old MSC cultured on young ECM, old ECM, and without ECM. Values are expressed as mean ± SD (two donors/group, three independent experiments/donor); *p < 0.05, **p < 0.01; Scale bars, 100 μm.

Confluent cultures of MSC from old and young donors were decellularized according to previously published procedures (Carvalho et al., 2019b, c; Silva et al., 2020; referred to as old ECM and young ECM, respectively). Before decellularization, both young MSC and old MSC were staining positive for collagen I, laminin, and fibronectin (Figure 4A). After decellularization, DAPI staining demonstrated only a residual amount of cellular nuclei, indicating that most of the cellular nuclei were removed (Figure 4B). Moreover, tissue culture plates displayed an ECM coating of fibrillar architecture staining positive for collagen I, laminin, and fibronectin (Figure 4B).

To determine the impact of donor age on the *in vitro* proliferation support capacity of MSC-derived ECM, MSC from young and old donors were cultured on decellularized young and old ECM. Cell numbers increased over time for young and old MSC cultured on all ECM, regardless of their origin (old or young ECM). Furthermore, Figures 4C,D suggest a beneficial cell response triggered by the presence of an ECM.

Concerning younger donors, after 7 days of cell culture, results showed that young MSC seeded on young ECM presented higher cell numbers [(9.37 ± 0.64) × 10^4^ cells, fold expansion of 7.8] than those cultured on old ECM (*p* < 0.05) or without ECM [(6.87 ± 0.98) × 10^4^ and (7.55 ± 1.05) × 10^4^ cells, respectively, and fold expansion of 5.7 and 6.3, respectively]. Furthermore, after 10 days of culture, young MSC cultured on young and old ECM demonstrated statistically significantly higher cell numbers (*p* < 0.05 for old ECM and *p* < 0.01 for young ECM) compared with cells cultured without ECM. These results suggest that MSC-derived ECM promoted a higher cell proliferative activity *in vitro*, regardless of the cell donor age (Figure 4C).

Interestingly, in what concerns older donors, after only 2 days of culture, old MSC presented a statistically significant increase in cell number when cultured on young ECM compared to old ECM and without ECM (*p* < 0.05; Figure 4D). Furthermore, young ECM was able to enhance the proliferative capacity of old MSC during the subsequent time points (day 4, 7, and 10). In fact, after 10 days, old MSC cultured on young ECM presented a statistically significant increase in cell numbers [(9.55 ± 0.91) × 10^4^ cells, fold expansion of 8] compared to old MSC cultured on old ECM [(6.80 ± 0.70) × 10^4^ cells, fold expansion of 5.7] or without ECM [(6.94 ± 0.59) × 10^4^ cells, fold expansion of 5.8] (*p* < 0.05). Instead, old ECM was not able to improve the proliferative capacity of old MSC.

### Effects of Donor Age on the Osteogenic Supportive Capacity of Mesenchymal Stromal Cells-Derived Extracellular Matrix

**FIGURE 5:**
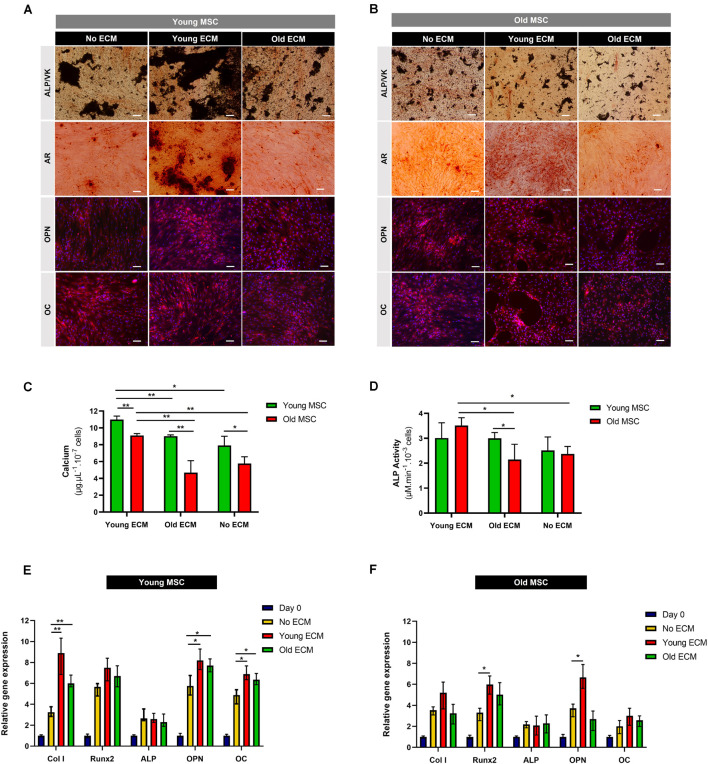
Impact of donor age on the osteogenic potential of mesenchymal stromal cell-derived extracellular matrix. **(A)** Alkaline phosphatase (ALP), von Kossa (VK), Alizarin Red, osteopontin (OPN), and osteocalcin (OC) stainings of young mesenchymal stromal cells (MSC; 30 and 45 years old) after 21 days of culture under osteogenic conditions on young extracellular matrix (ECM), old ECM, and no ECM. **(B)** ALP, VK, Alizarin Red, OPN, and OC stainings of old MSC (60 and 80 years old) after 21 days of culture under osteogenic conditions on young ECM, old ECM, and no ECM. DAPI was used to counterstain the cell nuclei in blue. DMEM: cells before differentiation (day 0), OSTEO: cells after 21 days of culture under osteogenic differentiation conditions. **(C)** Calcium deposition quantification of young and old MSC after 21 days under osteogenic differentiation conditions. **(D)** ALP activity of young and old MSC after 21 days under osteogenic differentiation conditions. **(E)** Osteogenic gene expression by young MSC cultured on young ECM, old ECM, and no ECM after 21 days under osteogenic differentiation conditions (*Col I*, *Runx2*, *ALP*, *OPN*, *OC*). **(F)** Osteogenic gene expression by old MSC cultured on young ECM, old ECM, and no ECM after 21 days of osteogenic differentiation (*Col I*, *Runx2*, *ALP*, *OPN*, *OC*). Results are normalized to the endogenous control GAPDH and presented as fold change expression relative to undifferentiated MSC (day 0). Values are expressed as mean ± SD (two donors/group, three independent experiments/donor); *p < 0.05, **p < 0.01; Scale bars, 100 μm.

Mesenchymal stromal cells cultured under osteogenic conditions on young ECM presented increased calcium deposition compared to cells cultured on old ECM or with no ECM, as shown by Alizarin Red and von Kossa stainings (Figures 5A,B). Moreover, young MSC cultured on young ECM presented a statistically significant increase in calcium deposition compared with MSC cultured on old ECM (*p* < 0.01; Figure 5C).

Interestingly, when old MSC were cultured on young ECM, MSC were able to produce a similar amount of calcium as young MSC differentiated on old ECM (9.02 ± 0.14 vs. 9.10 ± 0.22 μg.μl^–1^.10^–7^ cells). Old MSC cultured on young ECM presented increased calcium deposits compared to old MSC cultured without ECM or on old ECM. Thus, these results suggest that ECM age is an important variable in what concerns production of calcium deposits. Concerning ALP activity, old MSC cultured on young ECM presented a statistically significant increase compared to cells cultured without ECM (*p* < 0.05; Figure 5D). Contrarily, old ECM did not enhance the ALP activity of old MSC. Indeed, the quantification of ALP activity corroborates the promoting effect of young ECM on the osteogenic potential of old MSC.

Importantly, enhancement in mineralization of old MSC was only observed when cells were cultured on ECM from young donors. These results demonstrate a possible benefit of young ECM to rescue the *in vitro* osteogenic potential of impaired MSC.

In order to provide insights on the mechanistic origin of such osteogenic enhancement in MSC triggered by exposure to young ECM, osteogenic marker genes were analyzed (*Col I*, *Runx2*, *ALP*, *OPN*, and *OC*; Figures 5E,F). After 21 days under osteogenic differentiation conditions, MSC from young and old donors were able to up-regulate the expression of *Col I*, *Runx2*, *ALP*, *OPN*, and *OC* compared to control cells (day 0). Specifically, a statistically significant enhancement of the gene expression of *Col I* (*p* < 0.01), *OPN* (*p* < 0.05), and *OC* (*p* < 0.05) was observed when young MSC were differentiated on young and old ECM compared to no ECM (Figure 5E).

Nonetheless, a statistically significant enhancement of osteogenic gene expression of *Runx2* and *OPN* was observed when old MSC were cultured on young ECM compared to cells cultured on old ECM or without ECM (*p* < 0.05; Figure 5F). Interestingly, only young ECM promoted enhancement of osteogenic gene expression of old MSC compared to cells cultured without ECM.

## Discussion

Aging is a complex process involving multiple mechanisms, orchestrated at different levels, that still remain to be elucidated (Kirkwood, 2005). Clinical studies have shown that aging is associated with bone loss (Zaim et al., 2012; Corrado et al., 2020) and loss of the osteogenic potential of MSC (Sethe et al., 2006; Stolzing and Scutt, 2006; Baker et al., 2015; Pignolo et al., 2021). In this context, for instance, Mueller and Glowacki (2001) observed a decline in the osteogenic differentiation potential of MSC from donors above 60 years old. Additionally, MSC from aged donors not only showed reduced differentiation potential but also showed reduced proliferative capacity (Ganguly et al., 2017; Fafián-Labora et al., 2019). Altogether, these factors can compromise the feasibility of autologous MSC-based therapies in geriatric patients. Of notice, aged MSC acquire a senescent phenotype (Li et al., 2017; Campisi et al., 2019; Liu et al., 2020), which affects their clinical outcomes since transplantation of MSC with a large proportion of senescent cells is less effective.

Furthermore, the increase of senescent MSC *in vivo* with aging (Liu et al., 2020) has a negative impact on the integrity of their microenvironment, influencing the adhesion, migration, proliferation, and differentiation of these cells. This microenvironment is composed by an ECM of collagens, adhesion proteins, proteoglycans, and growth factors, forming the stem cell niche (Fuchs et al., 2004; Moore and Lemischka, 2006). ECM and cells establish a synergistic relationship: ECM provides appropriate cues to cells while cells secrete molecules that influence the ECM composition (Novoseletskaya et al., 2020). Additionally, stem cell behavior is dependent on biophysical aspects of the microenvironment, such as tissue stiffness, which is regulated by ECM organization and composition (Engler et al., 2006). The stiffness of the extracellular microenvironment is altered during aging and disease (Gattazzo et al., 2014), presenting reduced capacity to communicate instructive cues to cells (Kurtz and Oh, 2012). With aging, humans present glycosylation and proteomic damage of ECM proteins and modifications of cell–matrix interactions (Brownlee, 1995; DiLoreto and Murphy, 2015). For instance, Carlson and Conboy (2007) have reported that human embryonic stem cells lose their regenerative capacity when exposed to aged ECM. Therefore, when considering the development of regenerative therapies, it would be important to develop strategies to mimic the *in vivo* healthy niche. Thus, by reestablishing a proper microenvironment, it could be possible to provide the missing cues that are essential for cell survival, proliferation, and differentiation. Decellularized tissues, in which the ECM is preserved, have been used in tissue engineering applications and have confirmed the important role of ECM in the regulation of cell functions (Song and Ott, 2011; Gattazzo et al., 2014).

Recently, we have reported that decellularized ECM derived from BM MSC significantly enhanced the proliferation and osteogenic potential of MSC (Carvalho et al., 2019b, c; Silva et al., 2020). These findings led us to evaluate the effect of donor age on the osteogenic supportive capacity of MSC-derived ECM. Our initial characterization of isolated human MSC from both young (30 and 45 years old) and old (60 and 80 years old) donors exhibited typical MSC morphology and phenotype after four passages. MSC expressed CD73, CD90, and CD105 and lacked the expression of CD34 and CD45 markers, as established in Dominici et al. (2006). However, different studies have reported that throughout culture, MSC can gradually lose their typical morphology, starting to gain an irregular and flat shape at high passages (passage 9 for young MSC and passage 5 for old MSC) rather than a spindle-shaped morphology (Zaim et al., 2012). When testing the *in vitro* proliferative activity and osteogenic potential of MSC, the age-related differences were evident when comparing cells from young to old donors. We observed a decrease in the proliferation rate of MSC with donor age, consistent with other studies (over 60 years old; Fickert et al., 2010; Zaim et al., 2012; Payr et al., 2020). The decrease of the proliferative potential of MSC isolated from older patients might be linked to oxidative stress, since aged cells can form apoptotic bodies and accumulate β-galactosidase with high levels of reactive oxygen species and nitric oxide (Kornicka et al., 2015). In fact, these authors observed decreased antioxidative protection in the group of older donors (Kornicka et al., 2015). Concerning the osteogenic commitment of MSC, our results have confirmed that, in the conditions tested, aged MSC decreased their osteogenic capacity *in vitro*. We observed reduced production of calcium deposits and decreased expression of osteogenic gene markers *Col I*, *Runx2*, *OPN*, and *OC*. In fact, Mueller and colleagues showed an age-related decrease on the osteogenic potential of human BM MSC based on a decrease of ALP activity and expression (Mueller and Glowacki, 2001). Surprisingly, we did not observe any changes in ALP activity and its mRNA expression when comparing cells from the young and old age groups.

In what concerns ECM production, young and old MSC seemed to have produced equivalent ECM with a fibrillar architecture and a web-like structure composed at least by collagen I, fibronectin, and laminin. ECM derived from MSC is composed of a complex network of proteins containing at least collagen type I, collagen type II, collagen type IV, fibronectin, biglycan, decorin, and laminin (Lai et al., 2010). Fibronectin and collagen I have been reported to induce chemotaxis and proliferation activity of MSC (Thibault et al., 2007; Lindner et al., 2010) and to promote the osteogenic differentiation of MSC (Sun et al., 2011).

Most importantly, our data showed that old MSC were able to restore the proliferative and osteogenic potential of the aged cells when exposed to ECM derived from MSC originated from young donors (young ECM). Interestingly, our results suggest that age-related changes in the stem/stromal cell niche may affect the osteogenic potential of MSC. In fact, when exposed to a young ECM, proliferation and osteogenic differentiation capacity of MSC were increased significantly regardless of the age of MSC donors. In contrast, aged MSC were not able to enhance their osteogenic properties when cultured on old ECM. To our knowledge, these are the first findings suggesting that ECM derived from cultured young MSC can rescue the proliferative and the osteogenic potential of aged human MSC *in vitro*. Consistent with our results, although in the murine context, Sun et al. (2011) showed increased bone formation and reduced intracellular levels of reactive oxygen species by MSC (from either young and old mice) cultured on young ECM, but not on old ECM. It is also noteworthy that these authors have reported that both young and old murine MSC cultured on old ECM produced more adipose tissue *in vivo*, suggesting that old ECM may change the cell differentiation path of MSC.

Overall, our results suggest that the microenvironment plays a crucial role in determining the quality and quantity of human MSC. We have demonstrated that the aging of ECM is determinant of the osteogenic differentiation of MSC, since young ECM can enhance the proliferative and osteogenic capacity of old MSC. We consider that old MSC may alter the composition of their ECM and thus old ECM cannot restore the compromised osteogenic ability of old MSC as young ECM can. The alteration of ECM composition might affect the interaction of ECM with growth factors and cells. Future studies should focus on the characterization of the interaction of ECM with cells by using microscopy-based techniques, such as traction force microscopy to measure forces at the cell–ECM interface, and advanced imaging and spectroscopy techniques to visualize the complex interaction between cells and ECM (Liu et al., 2017). In this context, Sun and colleagues have shown that MSC–ECM derived from young mice and old mice presented different compositions of collagen I and proteoglycans. Moreover, a recent study from our group has reported that MSC that lack OC and OPN, two of the most important bone ECM proteins, presented an impaired osteogenic potential by downregulating osteogenic gene expression markers and decreasing calcium production (Carvalho et al., 2020, 2021). In fact, loss of OC and OPN is associated with patients that have their ECM compromised due to old age and other diseases, such as osteoporosis. Thus, we anticipate that ECM derived from older donors might have lost some important structural proteins that might impair cell interaction and behavior. Further proteomic analysis, such as mass spectrometry-based strategies (Byron et al., 2013; Silva et al., 2019), should be performed to evaluate the differences in ECM derived from old and young MSC.

In this study, we demonstrated the impact of donor age on the proliferative and osteogenic capacity of MSC. We found that human MSC from older donors presented reduced proliferative and osteogenic capacities. Furthermore, we investigated, for the first time, the potential use of MSC-derived ECM from young donors to rescue the proliferative and osteogenic potential of aged MSC for bone tissue engineering applications for geriatric patients. Our results highlight the ability of the stem cell niche to regulate cell behavior and the importance of ECM as a key component of that niche.

By unveiling the regulatory process that controls cell activity, it will be possible to develop new therapeutic strategies for aging-related diseases, improving the quality of life of patients. In particular, this study provides new insights on the osteogenic supportive capacity of decellularized cell-derived ECM during aging. Although *in vivo* studies should be performed before any further clinical use, our results open new therapeutic possibilities for elderly patients with limited bone formation capacity who currently lack effective treatments. This work corroborates the hypothesis that recapitulating a young microenvironment by using decellularized ECM derived from young MSC may overcome the age-related decline in cell quality, in particular, the osteogenic potential of these cells. We suggest that young ECM might attenuate the effects of the aging of the stem/stromal cell niche. Overall, ECM derived from young MSC can produce a “rejuvenated” bone microenvironment with high osteogenic regenerative capacity and might enhance bone regeneration processes mediated by aged cells.

## Data Availability Statement

The raw data supporting the conclusions of this article will be made available by the authors, without undue reservation.

## Author Contributions

MC and CS conceived and designed the study and wrote this manuscript. MC, LA, and IB obtained and processed datasets. JC and CS provided suggestions and supervised the research. All authors contributed to the article and approved the submitted version.

## Conflict of Interest

The authors declare that the research was conducted in the absence of any commercial or financial relationships that could be construed as a potential conflict of interest.

## Publisher’s Note

All claims expressed in this article are solely those of the authors and do not necessarily represent those of their affiliated organizations, or those of the publisher, the editors and the reviewers. Any product that may be evaluated in this article, or claim that may be made by its manufacturer, is not guaranteed or endorsed by the publisher.

## Supplementary Material

Supplementary Figure 1 Characterization of mesenchymal stromal cells (MSC) obtained from different donors (young MSC: 30 and 45 years old; old MSC: 60 and 80 years old). **(A)** Multilineage differentiation analysis of MSC from different donors. Cells were cultured under osteogenic, chondrogenic, and adipogenic differentiation media for 21 days. Alkaline phosphatase and von Kossa stainings were performed for osteogenic differentiation. Alcian Blue staining was performed for chondrogenic differentiation. Oil Red O staining was performed for adipogenic differentiation. Scale bars, 100 μm. **(B)** Surface marker expression by MSC isolated from all different donors by flow cytometry. MSC were positive for CD73, CD90, and CD105 but negative for CD34 and CD45.
