# Supplementary figures and images for: Impact of Donor Age on the Osteogenic Supportive Capacity of Mesenchymal Stromal Cell-Derived Extracellular Matrix
Source: Front Cell Dev Biol. 2021 Oct 5;9:747521. doi: 10.3389/fcell.2021.747521 (PMC8523799; doi:10.3389/fcell.2021.747521)

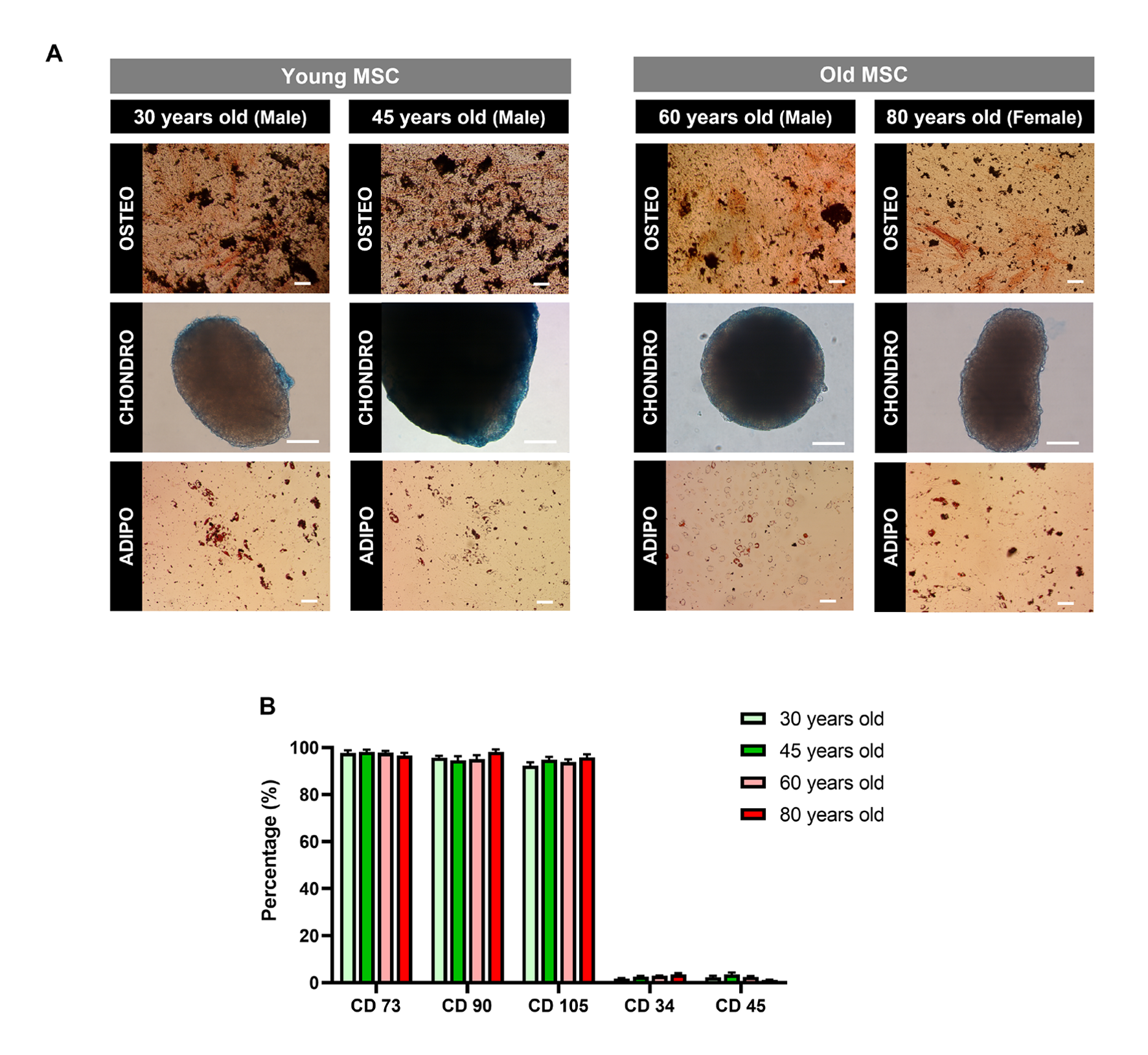

Supplement: Supplementary Figure 1 — Characterization of mesenchymal stromal cells (MSC) obtained from different donors (young MSC: 30 and 45 years old; old MSC: 60 and 80 years old). (A) Multilineage differentiation analysis of MSC from different donors. Cells were cultured under osteogenic, chondrogenic, and adipogenic differentiation media for 21 days. Alkaline phosphatase and von Kossa stainings were performed for osteogenic differentiation. Alcian Blue staining was performed for chondrogenic differentiation. Oil Red O staining was performed for adipogenic differentiation. Scale bars, 100 μm. (B) Surface marker expression by MSC isolated from all different donors by flow cytometry. MSC were positive for CD73, CD90, and CD105 but negative for CD34 and CD45. [file Image_1.PNG]
